# Evaluating within‐population variability in behavior and demography for the adaptive potential of a dispersal‐limited species to climate change

**DOI:** 10.1002/ece3.2573

**Published:** 2016-11-17

**Authors:** David J. Muñoz, Kyle Miller Hesed, Evan H. Campbell Grant, David A. W. Miller

**Affiliations:** ^1^ Department of Ecosystem Science and ManagementPennsylvania State UniversityUniversity ParkPAUSA; ^2^ Department of BiologyUniversity of MarylandCollege ParkMDUSA; ^3^ US Geological Survey Patuxent Wildlife Research CenterTurners FallsMAUSA; ^4^ Present address: Biology ProgramDepartment of Natural Sciences & MathematicsHesston CollegeHesstonKSUSA

**Keywords:** adaptive capacity, behavioral plasticity, climate change, color morph, demography, *Plethodon cinereus*

## Abstract

Multiple pathways exist for species to respond to changing climates. However, responses of dispersal‐limited species will be more strongly tied to ability to adapt within existing populations as rates of environmental change will likely exceed movement rates. Here, we assess adaptive capacity in *Plethodon cinereus*, a dispersal‐limited woodland salamander. We quantify plasticity in behavior and variation in demography to observed variation in environmental variables over a 5‐year period. We found strong evidence that temperature and rainfall influence *P. cinereus* surface presence, indicating changes in climate are likely to affect seasonal activity patterns. We also found that warmer summer temperatures reduced individual growth rates into the autumn, which is likely to have negative demographic consequences. Reduced growth rates may delay reproductive maturity and lead to reductions in size‐specific fecundity, potentially reducing population‐level persistence. To better understand within‐population variability in responses, we examined differences between two common color morphs. Previous evidence suggests that the color polymorphism may be linked to physiological differences in heat and moisture tolerance. We found only moderate support for morph‐specific differences for the relationship between individual growth and temperature. Measuring environmental sensitivity to climatic variability is the first step in predicting species' responses to climate change. Our results suggest phenological shifts and changes in growth rates are likely responses under scenarios where further warming occurs, and we discuss possible adaptive strategies for resulting selective pressures.

## 1 Introduction

Species' responses to shifting climate will be driven by a combination of ecological and evolutionary processes; however, predicting species' responses to climate change remains a challenging endeavor (Huey et al., 2012; Parmesan, 2006). Common methods for future range predictions ignore both the constraints, such as movement rates, and the adaptive capacity that influence how species respond to changes in their environment (Kearney & Porter, 2009; Thomas, Cameron, & Green, 2004). Multiple pathways exist for species to respond to climate change: dispersing into new habitats, evolving in response to changing conditions, ameliorating stressors via phenotypic plasticity, or going locally extinct (Lande & Shannon, 1996; Parmesan, 2006; Sinervo et al., 2010). While there is evidence that some species may be able to track their climate niche through time (Tingley, Monahan, Beissinger, & Moritz, 2009), it is unclear how less mobile species will respond to changing conditions. For dispersal‐limited species, including many amphibians (Gibbons et al., 2000; but see Smith & Green, 2005), environmental plasticity and evolution are crucial components of adaptive change because potential to respond in the near‐term through sufficient movement is limited, especially in fragmented landscapes (Cushman, 2006; Ruiz‐Aravena et al., 2014). Without dispersal, persistence will depend on the interplay of local demographic responses to climate and the degree to which negative responses can be minimized by plasticity and evolutionary adaptation.

At a basic level, predicting population persistence under climate change requires an understanding of the degree to which demography—growth, abundance, survival, recruitment, and emigration/immigration (Hanski & Gilpin, 1991; Thomas, 2000)—is influenced by environmental factors. Connecting climate change to shifts in demographic rates can be challenging (McCain, Szewczyk, & Bracy Knight, 2016), but there is growing evidence that climate change can alter vital rates for the worse, resulting in increased risk of population extinction (Barbraud & Weimerskirch, 2001; Rodenhouse, Christenson, Parry, & Green, 2009; Rohr & Palmer, 2012). For instance, Bestion, Teyssier, Richard, Clobert, and Cote (2015) showed experimental warming reduced adult survival in a common European lizard species. This resulted in lizard life history favoring earlier production of offspring in face of reduced life span. Despite this adaptive life‐history shift, a large portion of the species' populations are still predicted to go extinct before mid‐century (Bestion et al., 2015).

Phenotypic plasticity can mediate the response wildlife have to anthropogenic stressors (Hendry, Farrugia, & Kinnison, 2008). Behavioral plasticity, particularly in the timing, patterns, or extent of activity, is one of the most rapid phenotypic responses to novel conditions. These responses immediately affect the environmental conditions to which an individual is exposed (Snell‐Rood, 2013; Wong & Candolin, 2015). When responses are adaptive, behavioral plasticity can help a species or populations avoid the consequences of climate change. For example, some turtles are able to shift nest‐site selection to avoid detrimental warming conditions (Refsnider & Janzen, 2012). Behavioral responses can minimize short‐term impacts and may provide a mechanism for future adaptation via generation of novel traits (Gomez‐Mestre & Jovani, 2013; Zuk, Bastiaans, Langkilde, & Swanger, 2014).

The red‐backed salamander (*Plethodon cinereus*), a common North American woodland salamander, is an ideal model to examine these two components of adaptive capacity—demography and behavioral plasticity—in a dispersal‐limited species. *Plethodon cinereus* metapopulations exhibit minimal genetic exchange (Cabe et al., 2007; Marsh, Page, & Hanlon, 2008; Marsh et al., 2007). They are also able to modify the environmental conditions to which they are exposed both through horizontal movement for surface microhabitat selection and vertical movement between the surface and underground refugia (Heatwole, 1962; Jaeger, 1980; Spotila, 1972; Taub, 1961).

To evaluate within‐population variability, *P. cinereus* have a genetically inherited (Highton, 1959) color polymorphism that has previously been tied to differences in environmental tolerance. Although the direct mechanism remains elusive, this relationship between color and climate niche may be due to pleiotropy or linkage disequilibrium, but even the exact mode of inheritance is still unknown (Highton, 1959). Evidence suggests the two most common color polymorphisms—the striped morph and lead‐backed morph—respond differently to climatic drivers. Physiologically, lead‐backed morphs had lower metabolic rates compared to striped morphs (Moreno, 1989; Petruzzi, Niewiarowski, & Moore, 2006). Behaviorally, lead‐backed salamanders appear on the surface more during warmer temperatures, suggesting lower metabolic rates support higher tolerance of warm conditions (Anthony, Venesky, & Hickerson, 2008; Lotter & Scott, 1977; Moreno, 1989). Perhaps as a result of these differences, the frequency of both morphs varies geographically, with striped morphs more common in cooler, wetter regions and the lead‐backed more common in warmer, drier regions (Fisher‐Reid, Engstrom, Kuczynski, Stephens, & Wiens, 2013; Gibbs & Karraker, 2006; Lotter & Scott, 1977). Collectively, these studies suggest striped morphs prefer cool‐wet conditions and that lead‐backed morphs can better tolerate warm‐dry conditions. However, these climate–morph relationships have not been consistent (Petruzzi et al., 2006), and recent work has criticized the use of the color polymorphism for understanding climate relationships (Moore & Ouellet, 2015).

**Figure 1:**
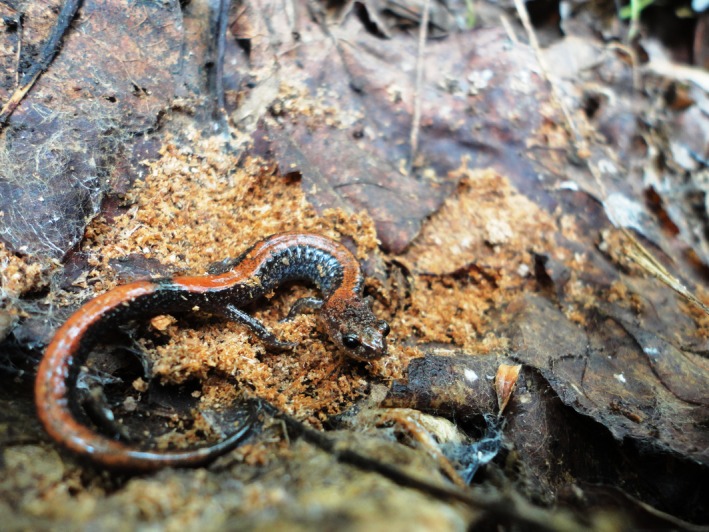
*Plethodon cinereus*, the red‐backed salamander, is a widely distributed and abundant woodland salamander in eastern North America

We use *P. cinereus* as a model organism for investigating within‐population variation in demography and behavioral plasticity in response to environmental conditions (Figure 1). As a proxy for within‐population variation in climate tolerance, we use the color polymorphism described above. Characterizing and understanding within‐population variation, particularly for traits tied to climate tolerance, should allow us to better understand the adaptive capacity of the species. Our goals were (1) to determine the extent to which *P. cinereus* surface activity and demography are impacted by environmental variation in temperature and precipitation and (2) to evaluate the validity of the color polymorphism as a mechanism for illuminating within‐population variation in climate response. We focus on the interaction between demographic and behavioral responses to environmental conditions. By simultaneously investigating plasticity and demography, we can improve predictions of how a species might adapt through within‐population variation, and we can use this relationship to determine how populations might be impacted by predicted climate change.

## 2 Materials and Methods

### 2.1 Data collection

Between October 2009 and May 2013, we conducted capture–mark–recapture surveys for *P. cinereus* at the Patuxent Wildlife Research Center (Laurel, MD, USA). Three plots were established >20 m apart in lowland‐deciduous hardwood forest under similar canopy conditions. Plots contained an array of cover boards (30.5 × 30.5 × 2.54 cm pieces of rough‐cut pine) spaced at 1‐m intervals, allowing us to effectively monitor *P. cinereus* populations and movement (Miller Hesed, 2012). Plot I was 20 × 20 m (400 cover boards), and plots II and III were 10 × 10 m (100 cover boards each). Captured salamanders were given individually identifying marks with visual implant elastomer, a technique that provides easily interpretable, long‐lasting marks (Gillette & Peterson, 2001; Grant, 2008). Gender, color morph (striped or lead‐back), and snout‐to‐vent length (SVL) were recorded every encounter and were independently determined twice to account for observer error. Environmental conditions (i.e., temperature, rainfall) were gathered from a weather station less than one kilometer away (2009–2012) or from a weather station three kilometers (2012–2013) from the study area. Although this reduced the resolution of our environmental data, the magnitude and direction of changing environmental conditions are highly correlated at such small spatial scales. We opportunistically surveyed plots 3–9 times each spring and autumn, ensuring a minimum of a week between surveys to maximize cover board effectiveness (Marsh & Goicochea, 2003). Plot I was surveyed from autumn 2009 to spring 2011, and plots II and III were surveyed from autumn 2009 to spring 2013.

We measured two types of responses: behavioral plasticity in the timing, duration, and extent of surface use during the spring and autumn and demography, including the rates of individual growth and population survival. Our general approach allowed us to determine (1) the degree to which behavior and demography responded to environmental variability and (2) whether the two color morphs differed in their response in concordance with past research (Table 1). To gain inference on our two responses, we used three quantitative approaches including mark–recapture (behavior and survival), spatial capture–recapture (movement), and nonlinear growth models (individual growth).

**Table 1:** Predictions generated by climate–morph relationships in the literature

Number	Factor	Climate–morph relationship prediction	Model parameter
Behavioral plasticity			
1	Surface use and timing	Striped: emerge earlier in spring, peak surface use in early spring, retreat earlier into summer, emerge later in autumn, peak surface use later in autumn, and retreat later in winter. Lead‐backed: emerge later in spring, peak surface use later in spring, retreat later into summer, emerge earlier in autumn, peak surface use later in autumn, and retreat earlier into winter	*p*, detection as function of calendar day
2	Surface use and water	Striped: surface use higher under wetter surface conditions. Lead‐backed: surface use higher under drier surface conditions	*p*, detection as function of rainfall
3	Surface use and temperature	Striped: surface use lower under warmer surface conditions. Lead‐backed: surface use lower under cooler surface conditions	*p*, detection as function of soil temperature
4	Breadth of surface movement	Striped: movement greater in cool/wet season (spring). Lead‐backed: movement greater in dry/warm season (autumn)	σ, breadth of surface use
Demography			
5	Seasonal survival	Striped: survival higher overwinter and lower over‐summer. Lead‐backed: higher over‐summer survival and lower overwinter survival	Φ, survival probability
6	Survival and temperature	Striped: survival lower under warmer temperatures and higher under cooler temperatures. Lead‐backed: survival higher under warmer temperatures and lower under cooler temperatures	Φ, survival probability
7	Seasonal growth	Striped: growth greater in winter and spring than lead‐backed. Lead‐backed: growth greater in summer and autumn than striped	*K*, growth coefficient
8	Growth and temperature	Striped: growth decrease under warmer temperatures. Lead‐backed: growth decrease under cooler temperatures	*K*, growth coefficient

Predictions one through four relate to morph differences in behavioral plasticity, and predictions five through eight relate to differences in demography. Predictions are based on evidence that the striped morph is cool‐wet‐adapted and the lead‐backed morph is warm‐dry‐adapted. For each prediction, a specific model was developed to test the effect of color morph, and the relevant parameter from that model is specified.

### 2.2 Behavioral plasticity analyses

*Plethodon cinereus* in our population are largely underground during the summer and winter due to unfavorable environmental conditions (Taub, 1961). Even during peak surface activity, *P. cinereus* may be unavailable for capture because they retreat to underground refugia (Bailey, Simons, & Pollock, 2004). We used robust design models that estimate within‐season detection probabilities and among‐season survival rates while accounting for this temporary unavailability (Kendall, Nichols, & Hines, 1997; Pollock, 1982). Detection probabilities reflect both the probability an individual was on the surface and the probability it was captured and identified. We can therefore estimate when, or under what conditions, salamanders are more likely to be on the surface and how this probability varies within each spring and autumn. Detection probability, parameter *p*, was modeled using a quadratic function to estimate the optimal environmental condition under which surface use peaked. We tested whether optima were different between morphs for three variables (Table 1): calendar day (prediction 1), the 3‐day average rainfall (prediction 2), and the 11‐day average of air temperature, which roughly characterizes surface soil temperature (prediction 3; Kang, Kim, Oh, & Lee, 2000). This resulted in three separate models, one for each environmental predictor. These models included a fixed effect of morph, a fixed effect for morph–environment interactions, and a fixed effect for plot to account for site differences (Table 2). Parameters were estimated using closed‐population robust design models in program MARK (White & Burnham, 1999). See Appendix A for further details on model development.

**Table 2:** Description of all models used to test the eight predictions about behavioral and demographic climate–morph relationships (Table 1)

Prediction number	Model description
1	*p*(plot + morph + calendar day × morph + calendar day^2^ × morph)
2	*p*(plot + morph + soil temperature × morph + soil temperature^2^ × morph)
3	*p*(plot + morph + rainfall × morph + rainfall^2^ × morph)
4	σ(morph)
5	Φ(plot + season × morph)
6	Φ(plot + temperature × morph)
7	*K*(season × morph)
8	*K*(season × temperature × morph)

Parameters are a function of the predictors found within parentheses. All predictors are fixed effects. Parameters not central to predictions found in Table 1 are not included but may be found in the Appendix D. Parameter *p* is detection probability, Φ is survival, σ is spatial breadth of movement, and *K* is growth coefficient.

Another aspect of behavioral plasticity is the breadth and extent of horizontal surface use. Salamanders move to forage, find mates, and defend their territories (Petranka, 1998). We investigated whether morphs moved differently depending on season, with autumn being warmer and drier and spring being cooler and wetter. Spatial capture–recapture models extend traditional mark–recapture models to better estimate population density by accounting for individual movement (Royle, Chandler, Sollmann, & Gardner, 2014). These models do so using the location of capture events to estimate a spatial parameter, σ. Morphs that exhibited greater breadth in surface use will have a larger estimated σ. Therefore, we would predict σ to be larger for the lead‐backed morph in autumn and larger for striped morphs in the spring (prediction 4; Table 1). For location, we used the coordinate of the cover board under which a salamander was found (Muñoz et al., in press; Sutherland, Muñoz, Miller, & Grant, 2016). We ran the spatial capture–recapture model separately for each season, using program R and package “runjags” to call program JAGS (Denwood, 2016; Plummer, 2003; R Core Team, 2014). For details, see Appendix B.

### 2.3 Demographic analyses

Closed‐population robust design models also estimate apparent survival probabilities (are alive and do not permanently emigrate from study site), Φ, among seasons while accounting for temporary emigration (Kendall et al., 1997; Pollock, 1982). To test predictions relating to demography and environmental conditions (prediction 5 and 6; Table 1), morph‐specific survival rates were modeled as a function of season and temperature (Table 2). Prediction 5 predicts that lead‐back morphs would have higher relative over‐summer survival and striped morphs would have higher relative overwinter survival. For prediction 6, we estimate how each morph's seasonal survival relates to mean summer temperature (mean low daily temperature for July and August) and mean winter temperature (mean low temperatures during January and February).

We modified the Faben's (1965) capture–recapture formulation of the von Bertalanffy growth model to estimate individual growth rates of SVL for each color morph (Schofield, Barker, & Taylor, 2013). Snout‐to‐vent length is a standard measurement of growth (Leclair, Levasseur, & Leclair, 2006), as salamanders can gain or lose mass rapidly depending on water availability. These models estimate two parameters: a growth coefficient, *K*, and an asymptotic maximum size, *L*_inf_. We allowed growth coefficients to differ by a season by morph interaction (autumn, winter, spring, and summer; Table 2). We would expect the rate of growth in summer and autumn to be higher for lead‐backed morphs and rate of growth in winter and spring is to be higher for striped morphs (prediction 7; Table 1). We defined seasons the same across all 4 years, where spring (March 2–May 16) and autumn (September 6–December 4) contained all field surveys. Summer and winter were periods when no surveys occurred and when most salamanders were expected to be underground. To test prediction 8, we examined the relationship of growth rate during the surface‐active seasons, autumn and spring, to the previous summer's or previous winter's mean temperature, respectively. Winter and summer growth rates were related to the current season's mean temperature. This allowed us to measure impacts during the hottest and coldest periods of the year and to account for the thermal inertia that carries over into the next season. We predicted that lead‐backed morphs would grow faster under hotter conditions and that striped morphs would grow faster under cooler conditions. We fit growth models using program R and package “runjags” to call program JAGS (Denwood, 2016; Plummer, 2003; R Core Team, 2014). See Appendix C for model description and JAGS code.

## 3 Results

Over the eight field seasons of the study, we had 2,805 captures during 86 sampling occasions of *P. cinereus* and approximately 20 captures of nontarget species. We captured 346 salamander in plot I (114 unique individuals and 232 recaptures; 48% lead‐backed); 1,039 salamanders in plot II (249 unique individuals and 790 recaptures; 40% lead‐backed); and 1,420 salamanders in plot III (389 unique individuals and 1,031 recaptures; 39% lead‐backed). After removing individuals that were only captured once, our sample size for estimating growth included 2052 observations for 479 individuals.

**Figure 2:**
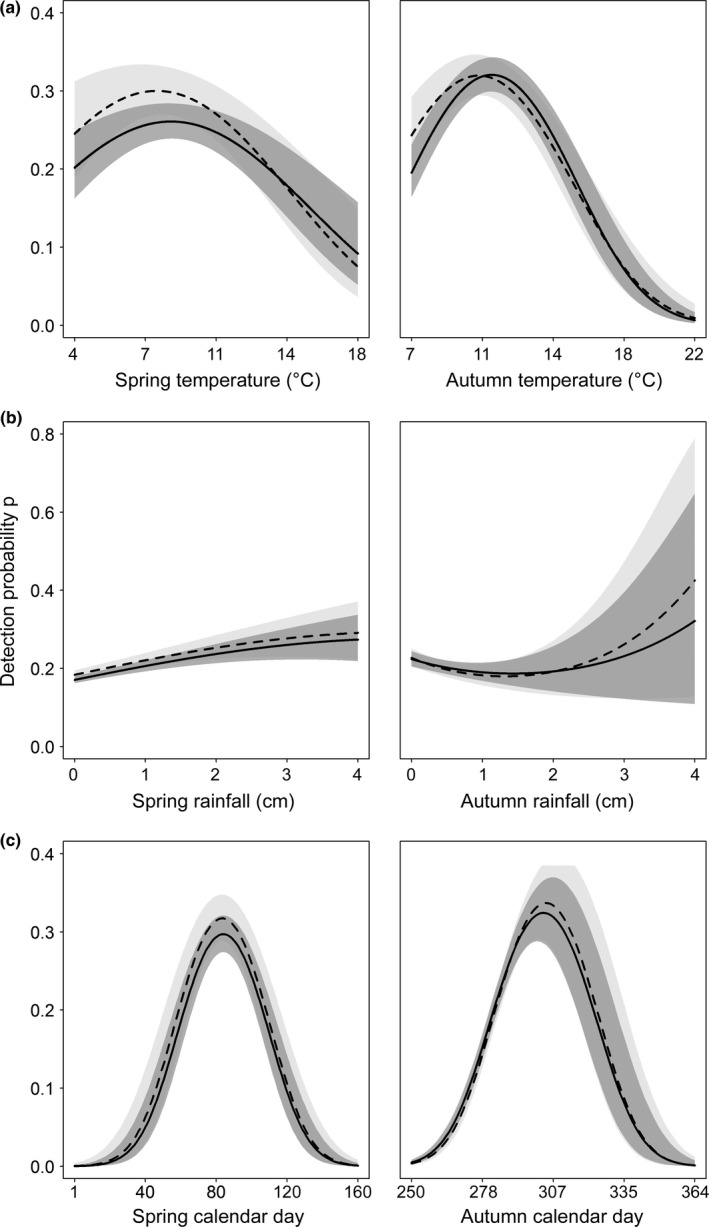
Surface detection as a function of soil temperature (a), rainfall (b), and calendar day (c) for *Plethodon cinereus* in Laurel, MD, USA. Spring (left) and autumn (right) detection functions are plotted for both morphs. Mean striped morph (solid) and mean lead‐backed morph (dashed) estimates are represented by lines. 95% confidence intervals are represented by shaded regions (striped = dark, lead‐backed = light). Both temperature and rainfall influence surface detection, leading to bimodal surface activity patterns

**Figure 3:**
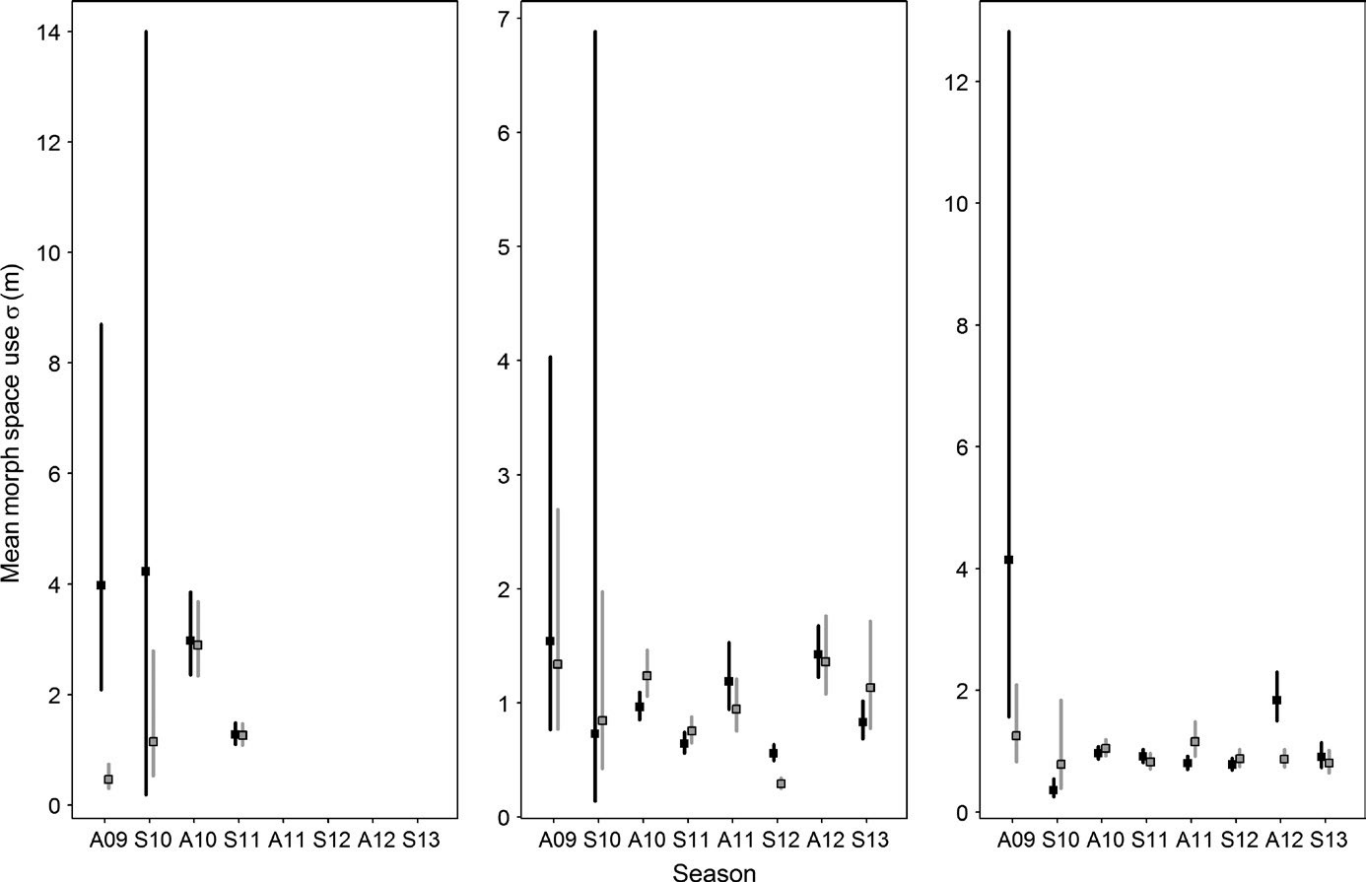
Breadth of surface space use across seasons (“A” autumn and year, “S” spring and year) for *Plethodon cinereus* in Laurel, MD, USA. Mean striped morph space use (black, with 95% Bayesian credible interval [BCI]) and mean lead‐backed morph space use (gray, with 95% BCI) do not exhibit any consistent patterns or trends between morphs. The first two seasons have large credible intervals, resulting from fewer survey occasions within those seasons

There were varying degrees of support for an effect of environmental conditions on salamander behavior. Striped and lead‐backed morphs maintained similar seasonal and environmental relationships, despite predictions otherwise (predictions 1–4). Detection probabilities peaked at intermediate temperatures (Figure 2a). Striped detection peaked at 11°C and lead‐backed individuals at 10.5°C in the autumn. In the spring, striped detection peaked at 8.5°C, and lead‐backed detection peaked at 8°C. Confidence intervals strongly overlapped however, suggesting no differences between morphs. For both morphs, detection increased with rainfall during the autumn and spring (Figure 2b). On average, both morphs had peak surface activity on March 25th in the spring and November 1st in the autumn (Figure 2c). We found weak support for breadth of surface activity to be greater in the autumn (Figure 3), but the 95% Bayesian credible interval for the two morphs overlapped in 17 of 20 of the plot–season combinations, indicating no strong movement differences between morphs. The largest mean breadth of movement was from striped morphs in plot I during the spring 2010 season (4.22 m ± 0.346 *SD*, 95% BCI [0.185, 14.0]), and the smallest was from lead‐backed morphs in plot II during the spring 2012 season (0.293 m ± 0.023, 95% BCI [0.251, 0.343]). Overall, mean striped breadth of movement across all combinations of plot and season was 1.55 m ± 1.24 *SD* and mean lead‐backed was 1.07 m ± 0.514 *SD*.

**Figure 4:**
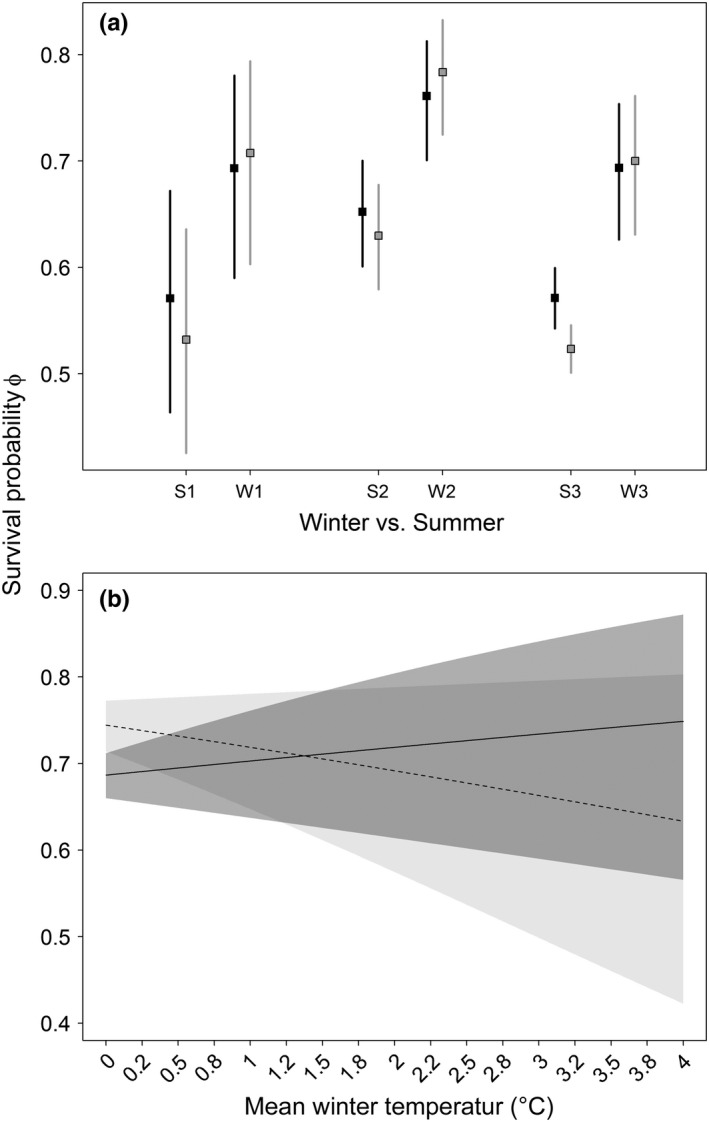
Estimates for overwinter (“W”) and over‐summer (“S”) survival probabilities in all three plots (“1”, “2”, and “3”; a) and survival probability as a function of mean winter temperature (b) for *Plethodon cinereus* in Laurel, MD, USA. (a) For plots 2 and 3, there were differences between summer and winter survival. Across plots and seasons, there were no clear differences between morphs (striped = black, lead‐backed = gray; squares = means, segments = 95% confidence intervals). (b) Striped mean survival (solid, with 95% CI) is not different from lead‐backed mean survival (dashed, with 95% CI). Both morphs exhibit relationships not different from zero. There was little summer variation in temperature, so no figure is provided

We found evidence that environmental conditions may affect survival and growth. Overwinter survival was generally higher than over‐summer survival across the three plots (Figure 4a). There were no significant differences between morphs, but simple comparisons of mean estimates suggest higher overwinter survival by lead‐back morph and higher over‐summer survival by the striped morph, contradicting prediction 5 (Table 1). We did not find support for a strong effect of temperature on survival probabilities for the population (Figure 4b). Warmer temperatures had a mean positive effect on striped morphs (winter: 0.355 ± 0.416 *SE*, 95% CI [−0.461, 1.17]; summer: 4.80 ± 5.78 *SE*, 95% CI [−6.53, 16.1]) and a mean negative effect for lead‐backed morphs (winter: −0.602 ± 0.414 *SE*, 95% CI [−1.41, 0.21]; summer: −2.09 ± 8.93 *SE*, 95% CI [−19.6, 15.4]). Mean trends contradict prediction 6, but all credible intervals overlapped zero, indicating no strong relationships.

**Figure 5:**
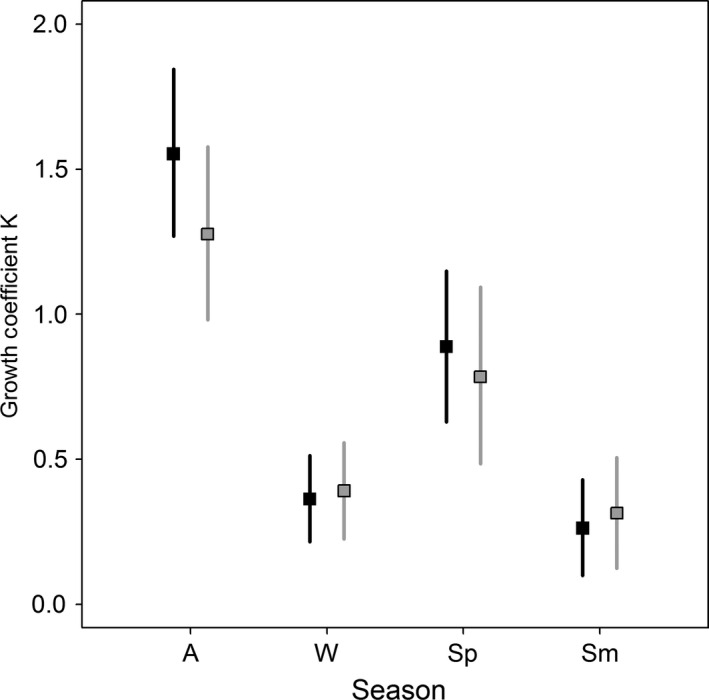
The predicted seasonal growth coefficients from the von Bertalanffy growth analyses for *Plethodon cinereus* in Laurel, MD, USA. Striped mean growth (solid black) is not different from lead‐backed growth (gray) in any of the four seasons (“A” autumn, “W” winter, “Sp” spring, and “Sm” summer). Means are presented as squares with 95% Bayesian credible intervals as segments

**Figure 6:**
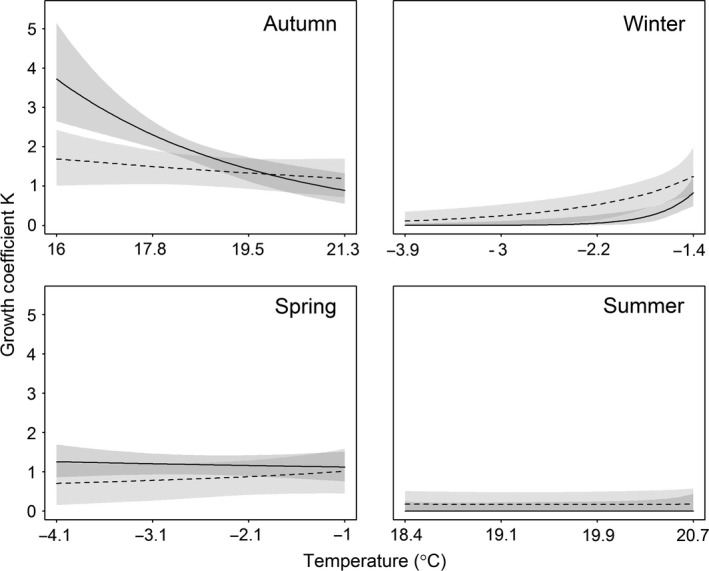
The impact of temperature on the growth coefficient for *Plethodon cinereus* in Laurel, MD, USA. In the autumn, mean growth declines as the previous summer's temperature increases for striped morphs (solid) and lead‐backed morphs (dashed). In the winter, increases for both morphs. In the spring, there were no strong relationships. Lastly, summer growth remained constantly low despite warming temperatures. Means are lines with 95% Bayesian credible intervals as shaded regions (striped = dark, lead‐backed = light)

Striped and lead‐backed morphs showed the fastest growth during the autumn, followed by less rapid growth in spring. In the winter and summer, growth was severely depressed in both morphs (prediction 7; Figure 5, Table 3). Warmer summer temperatures were negatively related to growth during the autumn, and striped morphs were more sensitive to warmer temperatures (striped β_temp_ = −0.588 ± 0.151 *SE*, 95% BCI [−0.890, −0.300]; lead‐backed β_temp_ = −0.143 ± 0.138 *SE*, 95% BCI [−0.416, 0.125]). Warmer winter temperatures did not influence growth rates in the spring for either morph (striped β_temp_ = −0.055 ± 0.133 *SE*, 95% BCI [−0.318, 0.202]; lead‐backed β_temp_ = 0.211 ± 0.354 *SE*, 95% BCI [−0.287, 0.927]). During the winter, warmer temperatures increased growth rates, particularly for striped morphs (striped β_temp_ = 4.885 ± 1.88 *SE*, 95% BCI [1.55, 8.86]; lead‐backed β_temp_ = 1.69 ± 0.964 *SE*, 95% BCI [0.679, 4.34]), whereas summer growth remained low regardless of temperature (striped β_temp_ = −1.75 ± 7.43 *SE*, 95% BCI [−19.1, 10.8]; lead‐backed β_temp_ = −1.11 ± 5.04 *SE*, 95% BCI [−15.5, 8.28]; Figure 6). Only prediction 8 was supported given that warmer temperatures more negatively impacted striped morphs in the autumn.

**Table 3:** Results from the von Bertalanffy growth models

Parameter	Description	Estimate
*K*_AL_	Autumn lead‐backed growth coefficient	1.28 ± 0.155 [0.981, 1.58]
*K*_AS_	Autumn striped growth coefficient	1.55 ± 0.146 [0.1.27, 1.84]
*K*_WL_	Winter lead‐backed growth coefficient	0.392 ± 0.085 [0.227, 0.557]
*K*_WS_	Winter striped growth coefficient	0.363 ± 0.075 [0.217, 0.513]
*K*_SpL_	Spring lead‐backed growth coefficient	0.784 ± 0.155 [0.486, 1.09]
*K*_SpS_	Spring striped growth coefficient	0.889 ± 0.133 [0.629, 1.15]
*K*_SmL_	Summer lead‐backed growth coefficient	0.315 ± 0.098 [0.124, 0.507]
*K*_SmS_	Summer striped growth coefficient	0.263 ± 0.084 [0.100, 0.430]
$L_{\inf_{L}}$	Asymptotic SVL (size) for lead‐backed	47.8 ± 0.506 [46.9, 48.9]
$L_{\inf_{S}}$	Asymptotic SVL (size) for striped	46.9 ± 0.348 [46.3, 47.6]
Temperature varying growth model, prediction eight		
β_0AL_	Mean autumn lead‐backed growth	0.338 ± 0.146 [0.020, 0.579]
β_0AS_	Mean autumn striped growth	0.625 ± 0.581 [0.475, 0.750]
β_0WL_	Mean winter lead‐backed growth	−1.22 ± 0.724 [−3.16, −0.406]
β_0WS_	Mean winter striped growth	−4.34 ± 1.49 [−7.58, −1.76]
β_0SpL_	Mean spring lead‐backed growth	−0.268 ± 0.380 [−1.10, 0.199]
β_0SpS_	Mean spring mean striped growth	0.160 ± 0.109 [−075, 0.353]
β_0SmL_	Mean summer lead‐backed growth	−4.41 ± 5.35 [−19.8, −0.693]
β_0SmS_	Mean summer striped growth	−7.91 ± 5.79 [−22.3, −1.52]
β_TempAL_	Autumn temperature effect on lead‐backed growth coefficient	−0.143 ± 0.138 [−0.416, 0.125]
β_TempAS_	Autumn temperature effect on striped growth coefficient	−0.588 ± 0.151 [−0.890, −0.300]
β_TempWL_	Winter temperature effect on lead‐backed growth coefficient	1.69 ± 0.964 [0.679, 4.34]
β_TempWS_	Winter temperature effect on striped growth coefficient	4.88 ± 1.88 [1.55, 8.86]
β_TempSpL_	Spring temperature effect on lead‐backed growth coefficient	0.211 ± 0.354 [−0.287, 0.927]
β_TempSpS_	Spring temperature effect on striped growth coefficient	−0.055 ± 0.133 [−0.318, 0.202]
β_TempSmL_	Summer temperature effect on lead‐backed growth coefficient	−1.11 ± 5.04 [−15.5, 8.28]
β_TempSmS_	Summer temperature effect on striped growth coefficient	−1.75 ± 7.43 [−19.1, 10.8]
$L_{\inf_{L}}$	Asymptotic SVL (size) for lead‐backed	47.9 ± 0.500 [47.1, 49.0]
$L_{\inf_{S}}$	Asymptotic SVL (size) for striped	47.1 ± 0.349 [46.4, 47.8]

Growth coefficient *K* determines the speed at which an individual grows. They were a function of both seasons (autumn, winter, spring, summer), seasonal temperature, and color morph. *L*_inf_ is the maximum size an individual can reach in Laurel, MD, USA. β represents coefficients from modeling growth as a function of seasonal temperature. Model parameters, parameter description, and the mean estimate (±*SE*, 95% Bayesian credible interval) are provided.

## 4 Discussion

Predicting species' responses to climate change require key data on a variety of aspects of an organism's ecology including both demography and behavior (Huey et al., 2012; Urban et al., 2016). Our study shows that the salamander population is clearly influenced by environmental and seasonal conditions both in use of surface habitat and in individual growth. Predicted climate change—warmer temperatures and more variable precipitation (Hayhoe et al., 2007)—will likely be detrimental to *P. cinereus* populations by shifting the timing and availability of suitable surface conditions. Additionally, we found that warmer temperatures dramatically reduce autumn growth, the most productive season for this species. We also attempted to characterize within‐population variation in behavior and demography, given it is a key mechanism for adapting to changing conditions (Barrett & Schluter, 2008). In our study system, we used a color polymorphism as a potential indicator of within‐population variation in climate change adaptive capacity. Multiple lines of evidence suggest that the *P. cinereus* color polymorphism may be linked to differences in climate niche; however, only one of our eight predictions, temperature‐dependent growth, provided moderate support for the climate–morph relationships. While some heterogeneity within the population can be explained by color morph, other ways of characterizing variation in climate tolerance are required.

Behaviorally, warmer temperatures and drier conditions both lead to a reduced presence on the surface for each morph (Figure 2a,b), likely leading to similar patterns in the timing of surface use (Figure 2c). Although none of the behavioral predictions were supported, contradicting past findings (Anthony et al., 2008; Fisher‐Reid et al., 2013; Lotter & Scott, 1977; Moreno, 1989), we did reveal that *P. cinereus* surface activity is strongly influenced by environmental variables. Two critical aspects of salamander ecology happen on the surface: foraging and courtship (Jaeger, 1980; Petranka, 1998). Our findings suggest that strong seasonal shifts to warmer and drier conditions may limit opportunities for *P. cinereus* surface activity. As a result, salamanders will need to change the timing of their use to find optimal conditions, increase their reliance on microhabitat refugia, or remain active on the surface under despite likely higher energetic costs (Homyack, Haas, & Hopkins, 2011).

Our seasonal estimates of survival show that mortality was generally greater during the summer than the winter (Figure 4a). In the southern portion of the *P. cinereus* range where our study takes place, it is likely that desiccation and heat stress in the summer is a greater driver of mortality than cold stress during the winter. Predation, breeding, and competition also likely contribute to the differences between summer and winter survival. Many salamander predators are in torpor during the winter (e.g., garter snake, *Thamnophis sirtalis;* Venesky & Anthony, 2007). *Plethodon cinereus* are also territorial, and antagonistic interactions for desirable microhabitat during the summer may impact demography, as they can result in the loss of a tail (Mathis, 1991; Schieltz, Haywood, & Marsh, 2010). Lastly, breeding may lower summer survival because clutch‐laying females may brood their clutch until their energy reserves are depleted (Yurewicz & Wilbur, 2004). Between morphs, we found slight evidence for mean lead‐backed summer survival to be lower than mean striped summer survival (Figure 4a). Rather than being climate driven, this is might be because lead‐back morphs have poorer quality diets (Anthony et al., 2008), are more submissive to striped morphs (Reiter, Anthony, & Hickerson, 2014), and have poorer quality territories (Paluh, Eddy, Ivanov, Hickerson, & Anthony, 2015). Striped morphs are also more territorial and aggressive, which may prevent lead‐backed morphs from finding necessary refugia during the summer (Reiter et al., 2014). When conditions become stressful during the summer, their survival may be negatively affected the most.

We found large differences in growth rates in relation to season and environmental conditions. Growth is highest in the autumn and is depressed when salamanders are underground in the winter and summer (Figure 5). Temperature variation during the winter and summer had clear impacts on salamander growth. Most importantly, hot summer temperatures affected growth in the autumn, with warmer summers decreasing growth by 1.5 times compared to our coolest summer observed (Figure 6). In the autumn, striped morphs responded more negatively to warming temperatures, following prediction 7. During the winter, both morphs responded positively to warmer temperatures. Warmer winter temperatures may increase the chances for opportunistic foraging (Caldwell & Jones, 1973). Contrariwise, hot summer temperatures reduce moisture availability and consequently the leaf‐litter invertebrate community (food for salamanders) and may force salamanders underground or to microhabitats where prey and suitable conditions persist (Jaeger, 1972, 1979). Further, hotter temperatures increase energetic costs, which can slow individual growth (Homyack, Haas, & Hopkins, 2010). Regardless of temperature, striped morphs had higher mean growth rates during surface‐active seasons (Figure 5), suggesting that other factors such as predator defense strategies, diet quality, or aggression and territoriality may play a larger role in individual growth than environmental preferences between morphs (Anthony et al., 2008; Paluh et al., 2015; Reiter et al., 2014; Venesky & Anthony, 2007).

Considering previous evidence for climate‐related differences in color morphs, it is surprising that only one of our predictions was supported. There are a few plausible reasons for this. First, *P. cinereus* populations are relatively isolated (Cabe et al., 2007), and it is possible that morph‐specific differences are not maintained in the population we studied. Differences would only be maintained if climate was a strong disruptive selective pressure (Barrett & Schluter, 2008), and Maryland's climate may be variable enough that selection is stabilizing. Behavioral adaptation is one of the fastest evolutionary responses, so stabilizing selection would result in a fixed breadth of behavioral responses for both morphs in a relatively short amount of time (Snell‐Rood, 2013). Second, the environmental conditions we observed may not have been extreme enough to influence demography, or if morph differences did exist, it may not lead to behavioral or demography differences for the factors we measured. Lastly, the *P. cinereus* color morph may not be a useful indicator in understanding climate tolerances. Our findings lend credence to growing evidence that the polymorphism is not tied to climate (Moore & Ouellet, 2015), but may be maintained by assortative mating (Anthony et al., 2008) or apostatic selection (Fitzpatrick, Shook, & Izally, 2009). Our study suggests that the color morph is an equivocal proxy at best for understanding climate tolerance variability.

One of the most coherent responses to climate change is shifts in species' range distributions (Parmesan, 2006). Dispersal‐limited species are less likely to exhibit range shifts and more likely must persist or witness range contractions (Midgley, Hughes, Thuiller, & Rebelo, 2006). Our results on the *P. cinereus* population may illuminate strategies for how dispersal‐limited species at large may persist. Behaviorally, our results add to the evidence that shifts in phenology, in order to match optimal conditions, are a likely response to climate change (Parmesan, 2006); however, the demographic costs to changes in phenology remain unexplored in many systems (Miller‐Rushing, Høye, Inouye, & Post, 2010). Our study suggests that even with possible changes in phenology, increasing summer temperatures will still likely reduce individual growth. Consequently, it could take longer for salamanders to become sexually mature (Nagel, 1977; Sayler, 1966).

Two adaptive strategies arise. First, selection will shift toward behavioral or physiological traits that ensure survival until reproductive size is reached (i.e., demographic buffering hypothesis; Boyce et al., 2006). Our results indicate resource availability will likely be restricted by future suboptimal conditions, so adaptive traits may be those that better secure resources such as suitable microhabitat. For many ectotherms, microhabitat can play an important role in buffering deleterious responses to climate change (Scheffers, Edwards, Diesmos, Williams, & Evans, 2014). However, the minimal variability in behavior in our population suggests physiological traits may become increasingly important. Other systems have shown physiological adaptation to warming conditions (spiders, Krehenwinkel & Tautz, 2013; plankton, Padfield, Yvon‐durocher, Buckling, Jennings, & Yvon‐durocher, 2015), but given the slower life history of *P. cinereus*, it is unlikely that adaptation can occur fast enough. Instead, thermal acclimation may play a central role in how the species mitigate climate‐driven restrictions in resource availability (Seebacher, White, & Franklin, 2014; but see Gunderson & Stillman, 2015).

A second adaptive pathway may select life‐history strategies that invest in reproduction at smaller sizes and younger ages. Variation in size at reproductive maturity already exists in many salamander populations (Peterman, Crawford, & Hocking, 2016; Tilley, 1973); however, “hastening” their life history, while providing more opportunities to reproduce, may reduce overall fecundity as smaller size correlates to fewer eggs per clutch in some salamander populations (Petranka, 1998). Other systems also show similar life‐history responses to climate change, including birds (Winkler, Dunn, & McCulloch, 2002), lizards (Bestion et al., 2015), and annual plants (Franks & Weis, 2008). Shifts in life history may be a key response to climate change for dispersal‐limited species, but it is unclear whether such a shift is sufficient for populations to persist.

Our goal was to understand how environmental conditions influenced behavior and demography and whether the color polymorphism was useful for understanding within‐population variability among those relationships. For organisms like *P. cinereus* that are dispersal‐limited, rapid environmental change may overwhelm plastic and adaptive pathways (Chevin, Lande, & Mace, 2010). While changes in populations and distributions are highly idiosyncratic across species (Gibson‐Reinemer & Rahel, 2015), our study suggests that projected increases in regional drought and temperature will act as strong negative environmental pressures on *P. cinereus* population persistence both behaviorally and demographically. Our results also show that the next step is to characterize genetic variability in responses. Genetic variability is a main driver of adaptive capacity (Barrett & Schluter, 2008), and although genomic resources relating genes to phenotypes for many species are undeveloped (Ekblom & Galindo, 2010), *P. cinereus* is widely studied and will likely have genomic data available in the near future. Our study provides necessary information and insights as to how *P. cinereus* will be impacted by future climate change (Huey et al., 2012; Urban et al., 2016; Williams, Shoo, Isaac, Hoffmann, & Langham, 2008) and suggests how it, and other dispersal‐limited species, may adaptively respond to such impacts.

## Conflict of Interest

None declared.

## Appendix A MARK Models

### A.1

#### A.1.1

Robust design population models use primary seasons (e.g., the autumn and spring seasons in our study) and secondary occasions (e.g., the survey occasions within seasons) to estimate abundance, apparent survival, detection, and temporary emigration (Kendall et al., 1997; Pollock, 1982). Within each season, populations are assumed to be closed. Probability of capture, *p*, and probability of recapture, *c*, are estimated and used to derive abundance. Between seasons, populations are open. During these intervals, the probability of moving outside the study area, γ″, the probability of staying outside the study area, γ′, and the apparent survival rate, Φ, are estimated. Bailey et al. (2004) showed that robust design models are best for estimating Plethodontidae demographic parameters.

Before testing predictions, we needed to account for some of the structure in the robust design models. First, what type of temporary emigration (γ′ and γ″ parameters) is most likely (Table A1)? For detection probabilities, is there a behavioral response to being trapped (trap happy or trap averse; *p, c* parameters; Table A2)? Lastly, how do we best account for temporal variation? Are parameters constant across time, additive, or interactive (Tables A3 and A4)? Below, we show the results from Akaike's information criteria (AIC_c_) model selection that determined the final structure of our models. Using the best‐supported structure for the nuisance parameters, we then generated five models to test predictions regarding differences in surface use and survival between the color morphs. This was carried out by examining support for an interaction between color morph and our driver of interest. This resulted in five models, each testing a different climate–morph prediction (predictions 1–3, 5, and 6; Table 2).

**Table A1:** Three basic emigration models were assessed

Model number	Model	Parameters	ΔAIC_c_	ω_*i*_	−2ln Likelihood
1	Random movement (γ′ = γ″)	148	0	0.977	14,291.4
2	Markovian movement (γ′ ≠ γ″)	159	7.485	0.023	14,274.3
3	No movement (γ′ = 1, γ″ = 0*)*	142	72.20	0	14,377.0

Random movement outperformed Markovian movement and no movement. To aid estimation, γ was set constant across all seasons, and thus, no other models are included here.

**Table A2:** We assessed whether or not salamanders exhibited a behavioral response to being captured

Model number	Model	Parameters	ΔAIC_c_	ω_*i*_	−2ln Likelihood
1	*p*(plot × survey occasion) = *c*(plot × survey occasion)	159	0	1.00	12,886.6
2	*p*(plot × survey occasion) ≠ *c*(plot × survey occasion)	429	461	0.0	12,671.5

Because the capture, *p*, and recapture probability, *c*, are modeled best when equal, there is no evidence of trap avoidance or “trap happy” behavior.

**Table A3:** Five survival models were assessed

Model number	Model	Parameters	ΔAIC_c_	ω_*i*_	−2lnLikelihood
1	Φ(plot + season)	24	0	0.735	14,999.7
2	Φ(plot + season + temp)	25	2.036	0.265	14,999.7
3	Φ(plot × season)	26	115.1	0	15,110.7
4	Φ(plot.)	18	171.5	0	15,183.4
5	Φ(plot + temp)	20	172.7	0	15,180.5

Plot effects were included in all analyses. Season represents overwinter and over‐summer survival. Temperature was the mean temperature of the months between primary sampling periods. Because models 1 and 2 were the most supported, it was clear that we could use them to test predictions 5 and 6.

**Table A4:** Second, we determined how detection varied across time

Model number	Model	Parameters	ΔAIC_c_	ω_*i*_	−2ln Likelihood
1	*p*(plot × survey occasion) = *c*	144	0	1.00	13,064.8
2	*p*(plot + survey occasion) = *c*	107	2,621	0.0	15,622.2
3	*p*(plot.) = *c*	30	3,695	0.0	16,858.6

The best model was interactive between plots and secondary sampling occasions. This allows us to test our predictions by modeling detection using secondary sampling occasion covariates like rainfall, temperature, and calendar day.

## Appendix B Model Used to Run SCR0 Bayesian Analysis with Color Morph Predictor in JAGS

### B.1

#### B.1.1

The most basic spatial capture–recapture model is referred to as SCR_0_ by Royle et al. (2014)_._ SCR_0_ is a single season closed‐population model for estimating density, and it can be used to estimate four parameters: abundance, density, detection probability, and breadth of movement. To estimate abundance, a homogenous binomial point process is used, where abundance, *N*, is a function of both all possible individuals, *M*, within the study area and animal density, ψ, so *N *~ Binomial (*M*, ψ). From the data, the model estimates the probability an unobserved individual belongs to the population (ψ), so SCR_0_ thins *M* possible individuals down to an estimate of abundance by $\hat{N}$ = *M*ψ. Density is simply $\hat{N}$ divided by the area of the study area.

The probability an individual will be captured at a given trap declines with distance from the animal's activity center, often specified using a half‐normal encounter probability,

(A1) $$p\left(x,s\right)=p_{0}\exp\left(-\frac{1}{2\sigma^{2}}\left||x_{j}-s_{i}|\right|\right),$$

where the probability of encountering an individual at location *x* with activity center *s* is a function of *p*_0_ the baseline detection probability, σ the breadth of the detection kernel, and ||*x*_*j*_ − *s*_*i*_|| the Euclidean distance between the location of trap *j* and the activity center of individual *i*. The spatial encounter histories (the data), *y*_*ijk*_, are then evaluated. The probability that individual *i* is caught (*y*_*ijk*_ = 1) or not caught (*y*_*ijk*_ = 0) in trap *j* on occasion *k* follows a Bernoulli trial where *y*_*ijk*_ ~ Bernoulli(*p*_*ijk*_), and *p*_*ijk*_ comes from equation (A1). In SCR_0_, detection is constant across the study period, so it is possible to “flatten” the encounter history so that captures (*y*_*ij*_ = 1, 2, 3, …, *n*) or noncaptures (*y*_*ij*_ = 0) represent the number of times an individual was caught in trap *j*, so *y*_*ij*_ **~ Binomial (*K, p*_*ij,*_) where *K* is the number of survey occasions, and *p*_*ij*_ is from equation (A1). Using logistic regression, we can reformulate equation (A1) into a new form that is convenient to add covariates of interest:

(A2) $$\log\text{it}\left(p_{ij}\right)=\alpha_{0}+\alpha_{1}\left|\right|x_{j}-s_{i}\left|\right|+\beta_{1}v_{1}+\cdots \beta_{t}v_{t},$$

where α_0_ is our baseline detection rate and α_1_ represents the coefficient for how fast detection decreases with distance. If we wanted to model detection or space use as a function of biological or environmental data, it is possible to estimate the effects (β_1_,* …*,* *β_*t*_) of *t* covariates (*v*_1_,* …*,* v*_*t*_) on detection.

As with other closed‐population abundance models, model SCR_0_ assumes demographic closure and some degree of geographic closure. However, SCR relaxes the assumptions of equal detectability among individuals. For instance, it is not necessary to ensure all individuals have a trap within their home range (“no holes”). Activity centers are assumed to be randomly distributed throughout the state space and are independent of each other. Given that this is rarely true in real animal populations, SCR models are fortunately robust to violations of the uniform distribution assumption. Third, detection is assumed to decline as distance increases from an animal's activity center. Lastly, encounters are assumed to be independent, meaning animals do not exclude one another and that individuals do not become adverse to traps.

We adapted equations (A1) and (A2) to a model in BUGS language to estimate parameters in a Bayesian framework. For the purposes of this study, we focused only on the estimation of σ, the spatial scaling parameter. We used vague priors for all parameters, and we took 36,000 total samples from the three Markov Chain Monte Carlo posterior distributions: 24,000 iterations with 1,000 burn‐in and thinning of 2. The three MCMC chains were visually assessed for convergence and accepted if the Gelman–Rubin statistic was <1.05 (Gelman & Rubin, 1992). Data were structured in a three‐dimensional matrix with axes individual *i,* trap location *j*, and survey occasion *K*. For more information on spatial capture–recapture models, see Muñoz et al. (in press).

#BUGS code to run SCR0 model in JAGS
model {
            #Priors psi ~ dunif(0,1)              #proportion of data augmented individuals
            psi.morph ~ dunif(0,1)        #proportion of striped morphs
            for(t in 1:2){                #loop over regression terms for each morph
                        alpha0[t] ~ dnorm(0,.1) 
                        logit(p0[t]) <- alpha0[t]
                        alpha1[t] <- 1/(2*sigma[t]*sigma[t])  #distance coefficient for logit function
                        sigma[t] ~ dunif(0, 15)
            }
            #Likelihood model
            for(i in 1:M){
                        z[i] ~ dbern(psi)	#probability z individuals are unobserved members of pop
                        Morph[i] ~ dbern(psi.morph)	#Probability of being striped
                        Morph.cat[i] <- Morph[i] + 1	#Convert to categorical variable
                        s[i,1] ~ dunif(xlim[1],xlim[2])	#distribution of activity centers
s[i,2] ~ dunif(ylim[1],ylim[2])
            for(j in 1:J){
                        d[i,j] <- pow(pow(s[i,1]-X[j,1],2) + pow(s[i,2]-X[j,2],2),0.5) #distance term
                        Y[i,j] ~ dbin(p[i,j],K)	#probability of encounter history of indy i at trap J at trap K
                        p[i,j] <- z[i]*p0[Morph.cat[i]]*exp(- alpha1[Morph.cat[i]]*d[i,j]*d[i,j]) 
                        #logistic regression probability
            }
}
            #Derived Parameters
            N <- sum(z[]) #abundance fxn of all probable individuals in state space
            N.stripe <- N*psi.morph
            N.unstripe <- N-N.stripe
            D <- N/900 # 30*30 area #Calculate density for the 30x30m state space
}

## Appendix C Growth Analyses and Models Used to Run von Bertalanffy Growth Bayesian Analysis in Program JAGS

### C.1

#### C.1.1

To estimate growth rates for each color morph, we modified the Faben's capture–recapture (1965) formulation of the von Bertalanffy growth model to estimate separate growth coefficients and asymptotic size for each color morph.

(A3) $${\hat{L}}_{r_{i}}=L_{m_{i}}+\left(L_{\inf_{i}}-L_{m_{i}}\right)\times \left(1-\exp\left(-K_{i}\times \left(\frac{\Delta_{i}}{365}\right)\right)\right),$$

where *i* is the interval between marking and recapture, ${\hat{L}}_{r_{i}}$ is the estimated size at recapture, $L_{m_{i}}$ is the size when an individual was marked, *L*_inf_ is the maximum size an individual can reach, *K*_*i*_ is the growth coefficient, and Δ*i* is the duration of the interval in days (scaled to year by dividing by 365). Only individuals captured more than once were used in this analysis, and SVL measurements were used for size.

To test the predictions regarding growth, we first modeled the growth coefficient as a function of four seasons to address prediction seven.

(A4) $${\hat{L}}_{r_{i}}=L_{m_{i}}+\left(L_{\inf_{i}}-L_{m_{i}}\right)\times \left(1-\exp\left(-Kf_{i}\left(\frac{\Delta f_{i}}{365}\right)-Kw_{i}\left(\frac{\Delta w_{i}}{365}\right)-Ksp_{i}\left(\frac{\Delta sp_{i}}{365}\right)-Ksm_{i}\left(\frac{\Delta sm_{i}}{365}\right)\right)\right),$$

where each of the growth coefficients represents the autumn, winter, spring, and summer, respectively. For each growth coefficient, the number of autumn season days, Δ*f*_*i*_, winter days, Δ*w*_*i*_, spring days, Δ*sp*_*i*_, and summer days, Δ*sm*_*i*_, were used for each capture interval. Seasons were defined the same across all 4 years, where the spring (March 2–May 16) and autumn (September 6–December 4) always contained all field surveys, and summer and winter were the periods between these surveys.

To test prediction eight, we modeled the growth as a function of mean season temperature. To evaluate the impacts of extreme heat and extreme cold, we modeled the surface‐active seasons (autumn and spring) as a function of previous summer and previous winter temperatures. Because the growth coefficient must always be positive to avoid estimation errors, the log of the growth coefficients was modeled as,

(A5) $$\log\left(K_{i}\right)=\beta_{0}+\beta_{\mathrm{temp}}\times T_{i},$$

where β_0_ is the mean growth rate in each season, β_temp_ is the coefficient for how growth changes with temperature, and *T*_*i*_ is the mean of the season's low temperature in interval *i*. Temperature effects were ran separately for each of the four seasons. The temperature coefficients will determine whether morphs exhibit differential demography in regard to heat or cool stress.

This model assumes that the growth models start from age zero and that growth rates are conditional on the maximum asymptotic size an animal can reach. We used vague priors for all parameters and kept 3,000 iterations from each of three chains after an initial burn‐in of 1,000 iterations. We visually assessed chains for convergence and that the Gelman–Rubin statistic was <1.05 (Gelman & Rubin, 1992). 
#BUGS model for testing prediction 7, the effect of seasonal growth.
model{
            model{
                for(i in 1:n){
                                     #Fabens mark–recapture formulation
                                     Lr[i]~dnorm(Lr.hat[i], tau.Lr)

                                     Lr.hat[i] <- Lm[i] + (L.inf[m[i]]-Lm[i])*(1-exp(-Ksp[m[i]]*(dSp[i]/365) + -Ksm[m[i]]*(dSm[i]/365) + -Kf[m[i]]*(dF[i]/365) + -Kw[m[i]]*(dW[i]/365)))

                        }
                        for(j in 1:J){
                                     L.inf[j]~dnorm(0,.001)
                                     Ksp[j]~dunif(0,5)
                                     Kf[j]~dunif(0,5)
                                     Ksm[j]~dunif(0,5)
                                     Kw[j]~dunif(0,5)
                        }
                #Priors 
                tau.Lr <- pow(sigma.Lr,-2)
                sigma.Lr ~ dunif(0,5)
}

#BUGS model for testing prediction 8, the effect of temperature on seasonal growth
model{
model{
            #Priors 
            for(j in 1:J){
                        L.inf[j] ~ dnorm(0,.001)
                        B0sp[j] ~ dnorm(0, 0.01)
                        Btsp[j] ~ dnorm(0, 0.01)
                        B0sm[j] ~ dnorm(0, 0.01)
                        Btsm[j] ~ dnorm(0, 0.01)
                        B0f[j] ~ dnorm(0, 0.01)
                        Btf[j] ~ dnorm(0, 0.01)
                        B0w[j] ~ dnorm(0, 0.01)
                        Btw[j] ~ dnorm(0, 0.01)
            }
            tau.Lr <- pow(sigma.Lr,-2)
            sigma.Lr ~ dunif(0,5)
            #Model Fabens mark–recapture formulation
            for(i in 1:n){
                        Lr[i] ~ dnorm(Lr.hat[i], tau.Lr)

                        Lr.hat[i] <- Lm[i] + (L.inf[m[i]]-Lm[i])*(1-exp(-Ksp[i]*(dSp[i]/365) + -Ksm[i]*(dSm[i]/365) + -Kf[i]*(dF[i]/365) + -Kw[i]*(dW[i]/365)))
                        log(Ksp[i]) <- B0sp[m[i]] + Btsp[m[i]]*tempSp[i]
                        log(Ksm[i]) <- B0sm[m[i]] + Btsm[m[i]]*tempSm[i]
                        log(Kf[i]) <- B0f[m[i]] + Btf[m[i]]*tempF[i]
                        log(Kw[i]) <- B0w[m[i]] + Btw[m[i]]*tempW[i]
            }
}

## Appendix D Final Eight Models to Test Climate–Morph Predictions

### D.1

#### D.1.1

Predictions 1–3 and 5–6 were analyzed in program MARK (White & Burnham, 1999). The remaining predictions were analyzed in a Bayesian framework using program R (R Core Team 2014), R package “runjags” (Denwood, 2016), and program JAGS (Plummer, 2003).

**Table A5:** Description of all models used to test the eight predictions about behavioral and demographic climate–morph relationships (Table 1)

Prediction number	Model description
1	Φ(plot + season + morph), γ′(morph.) = γ″(), *p*(plot + morph + calendar day × morph + calendar day^2^ × morph) = c()
2	Φ(plot + season + morph), γ′(morph.) = γ″(), *p*(plot + morph + soil temperature × morph + soil temperature^2^ × morph) = c()
3	Φ(plot + season + morph), γ′(morph.) = γ″(), *p*(plot + morph + rainfall × morph + rainfall^2^ × morph) = c()
4	*N*(morph) + *p*(morph) + σ(morph)
5	Φ(plot + season × morph), γ′(morph.) = γ″(), *p*(plot + morph + calendar day × morph + calendar day^2^ × morph) = c()
6	Φ(plot + temperature × morph), γ′(morph.) = γ″(), *p*(plot + morph + calendar day × morph + calendar day^2^ × morph) = c()
7	*K*(season × morph) + *L*_inf_(morph)
8	*K*(season × temperature × morph) + *L*_inf_(morph)

Parameters are a function of the predictors found within parentheses. Empty parentheses () indicate predictors are identical to the previous model parameter. Values set as constant are represented as (.)

**Table A6:** Results from MARK behavioral analyses (predictions 1–3)

Model	Parameters	ΔAIC_c_	ω_*i*_	−2ln Likelihood
1	26	0.0	1.0	14,972.2
2	26	165.8	0.0	15,138.0
3	26	563.7	0.0	15,535.9

The most likely model for explaining *Plethodon cinereus* surface activity has surface detection as a quadratic function of calendar day.
